# Two RmlC homologs catalyze dTDP-4-keto-6-deoxy-d-glucose epimerization in *Pseudomonas putida* KT2440

**DOI:** 10.1038/s41598-021-91421-x

**Published:** 2021-06-07

**Authors:** Franziska Koller, Jürgen Lassak

**Affiliations:** Department Biology I, Microbiology, Ludwig-Maximilians-Universität München, Planegg/Martinsried, Germany

**Keywords:** Microbiology, Molecular biology

## Abstract

l-Rhamnose is an important monosaccharide both as nutrient source and as building block in prokaryotic glycoproteins and glycolipids. Generation of those composite molecules requires activated precursors being provided e. g. in form of nucleotide sugars such as dTDP-β-l-rhamnose (dTDP-l-Rha). dTDP-l-Rha is synthesized in a conserved 4-step reaction which is canonically catalyzed by the enzymes RmlABCD. An intact pathway is especially important for the fitness of pseudomonads, as dTDP-l-Rha is essential for the activation of the polyproline specific translation elongation factor EF-P in these bacteria. Within the scope of this study, we investigated the dTDP-l-Rha-biosynthesis route of *Pseudomonas putida* KT2440 with a focus on the last two steps. Bioinformatic analysis in combination with a screening approach revealed that epimerization of dTDP-4-keto-6-deoxy-d-glucose to dTDP-4-keto-6-deoxy-l-mannose is catalyzed by the two paralogous proteins PP_1782 (RmlC1) and PP_0265 (RmlC2), whereas the reduction to the final product is solely mediated by PP_1784 (RmlD). Thus, we also exclude the distinct RmlD homolog PP_0500 and the genetically linked nucleoside diphosphate-sugar epimerase PP_0501 to be involved in dTDP-l-Rha formation, other than suggested by certain databases. Together our analysis contributes to the molecular understanding how this important nucleotide-sugar is synthesized in pseudomonads.

## Introduction

**Figure 1:**
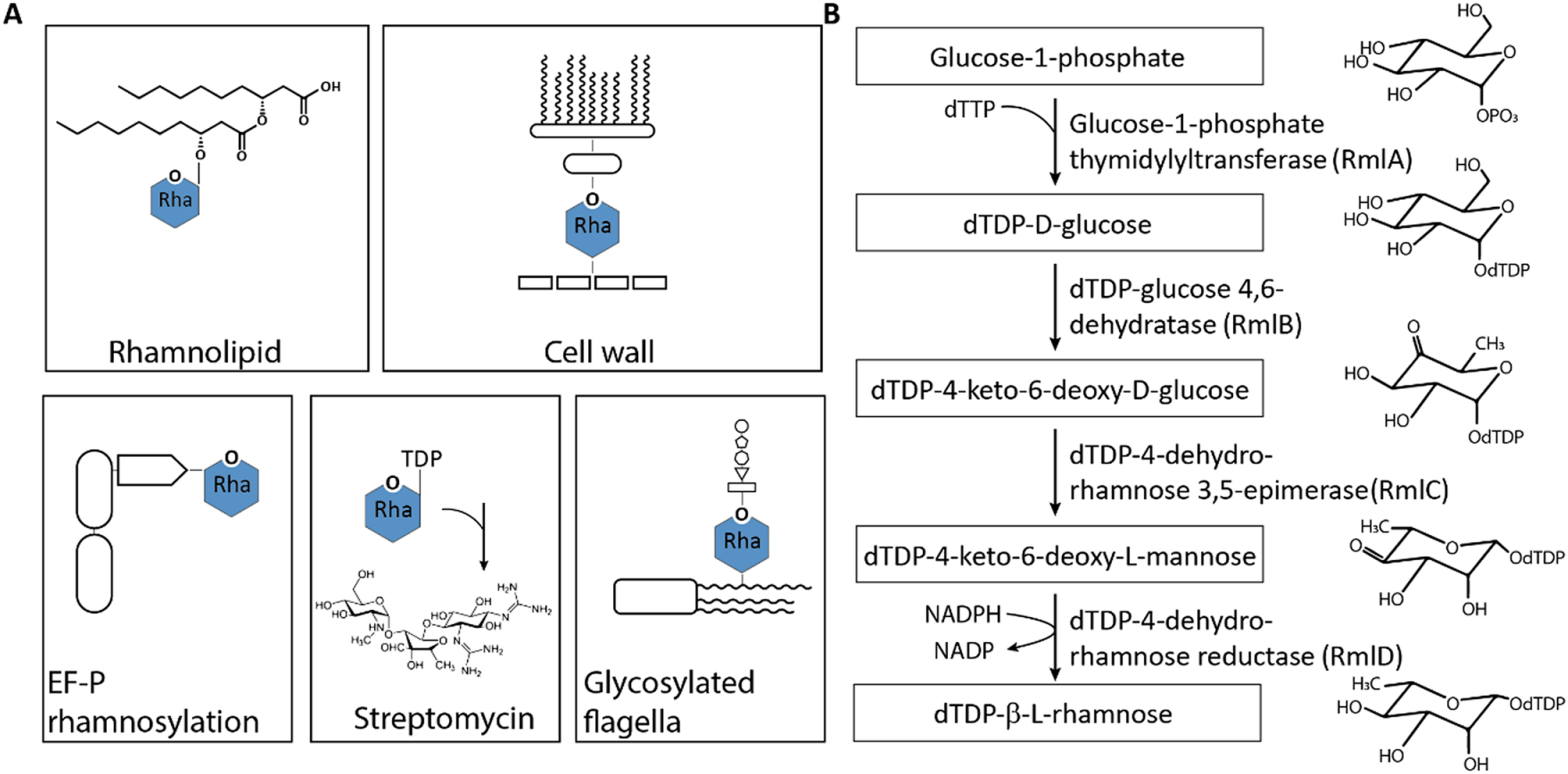
Rhamnose as versatile building block in composite biomolecules. (**A**) l-Rha in bacterial biomolecules. Top left: Rhamnolipids consisting of a rhamnose moiety and a fatty acid tail in *P. aeruginosa*^9^. Top right: Mycobacterial cell wall containing l-Rha as linking sugar between arabinogalactan and peptidoglycan^9^. Bottom left: Rhamnosylation of translation elongation factor EF-P in about 10% of all bacteria^6^. Bottom middle: biosynthesis of Streptomycin inter alia originating from dTDP-l-Rha^10^. Bottom right: Glycosylated flagella with a linking l-Rha moiety in certain *Pseudomonads*^11^*.* (**B**) dTDP-β-l-rhamnose biosynthesis pathway. Glucose-1-phosphate thymidylyltransferase, the first enzyme of the pathway, transfers a thymidylmonophosphate nucleotide to glucose-1-phosphate, which is further oxidated by dTDP-d-glucose 4,6-dehydratase at the C4 hydroxyl group of the saccharide. The double epimerization reaction at positions C3 and C5 is catalyzed by the dTDP-4-keto-6-deoxy-d-glucose 3,5-epimerase. Finally, the reduction of the C4 keto group by the dTDP-4-keto-6-deoxy-l-mannose reductase leads to dTDP-l-Rha.

Rhamnose (Rha) is a naturally occurring sugar being widely distributed among bacteria and plants^1^. Rha is a component of saponins^2^, certain bacterial glycans such as rhamnolipids^3^ or mycolic acids^4^, extracellular polysaccharides^5^ and even cytosolic proteins^6^ (Fig. 1A). Incorporation of rhamnose into these compounds requires an activated precursor which is provided as a nucleotide sugar. To date, two forms of activated Rha are known to be produced by bacteria: Guanosine diphosphate-α-d-rhamnose (alternative name: 6-deoxy-α-d-mannose) (GDP-Rha)^7^ and deoxythymidine-β-l-rhamnose (dTDP-l-Rha)^8^. While GDP-Rha is synthesized from mannose-1-phosphate^7^, the pathway for dTDP-l-Rha starts with glucose-1-phosphate (Glc-1P) (Fig. 1B).

Homologs for the synthesis genes of dTDP-l-Rha, *rmlBDAC*, can be identified in gram-positive and gram-negative bacteria^1^ and according to their number the pathway consists of four steps (Fig. 1B)^1^. First, a nucleotide transferase RmlA (also named RfbA^12^ or RffH^13^) transfers a deoxythymidine monophosphate moiety from deoxythymidine triphosphate to Glc-1P accompanied by the release of pyrophosphate. In the second step, a dehydratase RmlB (also named RfbB^14^ or RffG^13^) catalyzes the conversion of dTDP-glucose into dTDP-4-keto-6-deoxy-d-glucose. The third enzyme—an epimerase RmlC (also named RfbC^15^)—mediates a double epimerization reaction leading to the formation of dTDP-4-keto-6-deoxy-l-mannose. Fourth, RmlD (also named RfbD)^15^ reduces the C4 keto group of the 4-keto-6-deoxy-l-mannose and with this dTDP-l-Rha synthesis is completed. Notably, the pathway was shown to be critical or even essential for viability in the human pathogens *Streptococcus pyogenes*, *S. mutans*^16^ and *Mycobacterium tuberculosis*^17^. In the clinically relevant *Pseudomonas aeruginosa*^18^*,* dTDP-l-Rha is important for the synthesis of rhamnolipids^19^. These are bacterial surfactants with a rhamnose moiety as head group and act as a key virulence determinant^20^. Moreover, in about 10% of all bacteria including pseudomonads, a protein monorhamnosylation was described in 2015 which is essential for activation of the polyproline specific translation elongation factor EF-P^6^. Specifically, the glycosyltransferase EarP transfers a rhamnose moiety onto a conserved EF-P arginine residue R32 thereby utilizing dTDP-l-Rha as a precursor^6,21–23^. In the scope of this study, we investigated the dTDP-l-Rha biosynthesis pathway of *P. putida* KT2440 with focus on the epimerization of TDP-4-keto-6-deoxy-d-glucose. *P. putida* strains in general are fast-growing and genetically easily accessible^24^. They are a paradigm of metabolically versatile microorganisms being able to recycle organic wastes and are key players in the maintenance of environmental quality^24^.

Following an unbiased approach and utilizing a restriction based genomic library, we identified the two paralogous proteins PP_1782 (now termed RmlC1) and PP_0265 (now termed RmlC2) as dTDP-4-dehydrorhamnose 3,5-epimerases while the last step namely the reduction to dTDP-l-Rha seems to be solely catalyzed by PP_1784 (RmlD). By contrast, two further candidate genes that were identified by database mining and homology analyses—PP_0500 and PP_0501—are not involved in dTDP-l-Rha biosynthesis. Taken together, our findings contribute to the molecular understanding how dTDP-l-Rha is synthesized in *Pseudomonas putida* KT2440.

## Results

### A screening system that allows for the discovery of dTDP-l-Rha synthesis genes

**Figure 2:**
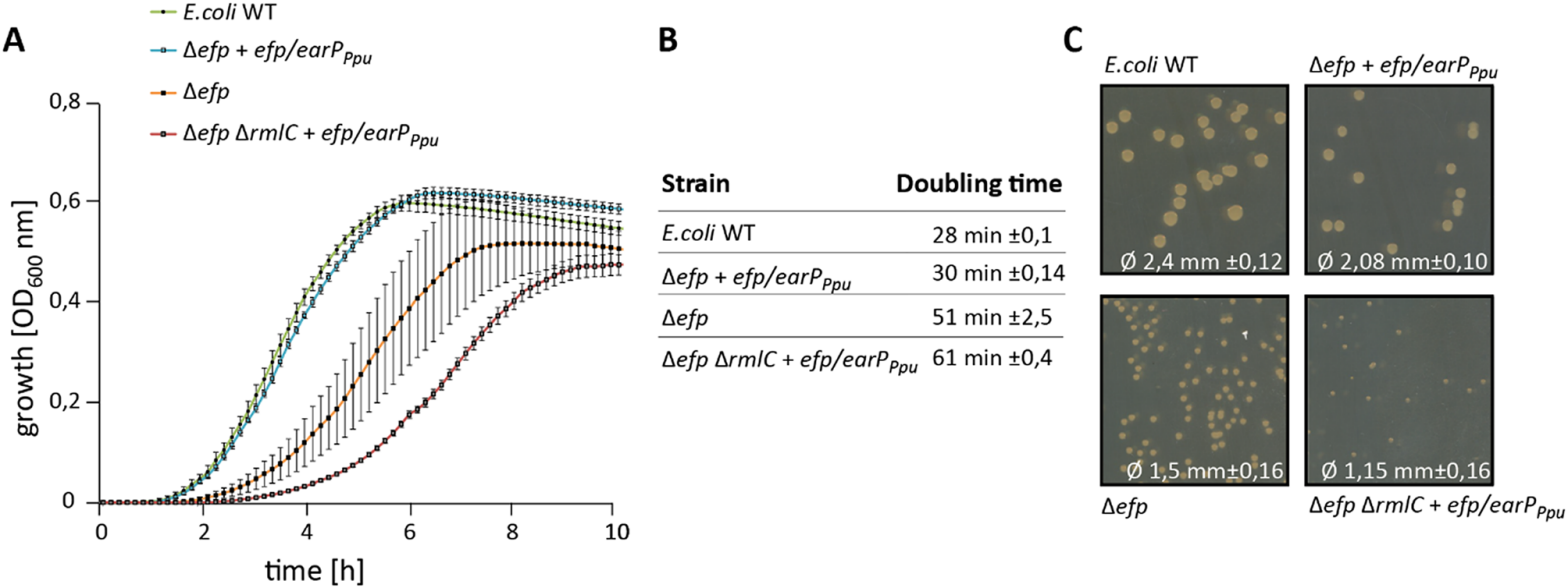
Growth analysis of cross complemented *E.coli* Δ*efp* mutants in dependence of the dTDP-l-Rha pathway. (**A**) *E. coli* MG1655 (*E. coli* WT), *E. coli* MG1655 Δ*efp* expressing pBBR MCS2 *efp*/*earP*_*Ppu*_ (Δ*efp efp*/*earP*_*Ppu*_), *E. coli* MG1655 Δ*efp* (Δ*efp*) and *E. coli* MG1655 Δ*efp* Δ*rmlC* expressing pBBR MCS2 *efp*/*earP*_*Ppu*_ (Δ*efp* Δ*rmlC efp*/*earP*_*Ppu*_) were grown in LB at 37 °C. Shown is a the mean curve from three independent biological replicates with standard deviations. (**B**) Doubling times of *E. coli* strains listed in A. Doubling times were calculated from growth analysis from three independent biological replicates. (**C**) Comparison of colony size. *E. coli* MG1655 (*E. coli* WT), *E. coli* MG1655 Δ*efp* expressing pBBR MCS2 *efp*/*earP*_*Ppu*_ (Δ*efp efp*/*earP*_*Ppu*_), *E. coli* MG1655 Δ*efp* (Δ*efp*) and *E. coli* MG1655 Δ*efp* Δ*rmlC* expressing pBBR MCS2 *efp*/*earP*_*Ppu*_ (Δ*efp* Δ*rmlC efp*/*earP*_*Ppu*_) were plated on LB Agar (1.5%). The mean diameter with respective standard deviations is depicted at the bottom. Pictures were taken after o/n growth at 37 °C.

To identify genes involved in dTDP-l-Rha biosynthesis, we took advantage of cross functionality of pseudomonal EF-P in *Escherichia coli* and the fact that activation of the translation factor strictly depends on the nucleotide sugar as donor substrate. This cannot necessarily be expected, as the *E. coli* endogenous EF-P significantly differs from its pseudomonal counterpart^25^: although both proteins alleviate ribosome stalling at polyproline stretches^6,26^, their modes of activation are phylogenetically unrelated^6,27^. While *E. coli* EF-P (EF-P_*Eco*_) strictly depends on (*R*)-β-lysylation^22,28–30^ and hydroxylation^31^ of a conserved lysine, *Pseudomonas* EF-P (EF-P_*Ppu*_) is rhamnosylated at an arginine by the glycosyltransferase EarP (EarP_*Ppu*_) at the structurally equivalent position^6,21^. Despite these apparent distinct post-translational modifications, a combination of *efp*_*Ppu*_ and *earP*_*Ppu*_ from *P. putida* KT2440 can compensate for a lack of *efp* in *E. coli* (Δ*efp*) as long as the endogenous dTDP-l-Rha pathway remains intact (Fig. 2A,C)^6^. Interestingly, loss of any synthesis gene—here exemplified with a Δ*rmlC* strain—does not simply phenocopy Δ*efp* but even results in more severe growth defects, as can be concluded from the corresponding doubling times (Fig. 2B): *E. coli* Δ*efp* cross complemented with *efp*/*earP*_*Ppu*_ grows twice as fast as the same strain additionally lacking *rmlC* (Δ*efp* Δ*rmlC*). These growth defects are also reflected by the size of the colonies (Fig. 2C). The differences in growth rates provide us with a selection regime to identify dTDP-4-dehydrorhamnose-3,5-epimerase genes from a *P. putida* genomic library.

**Figure 3:**
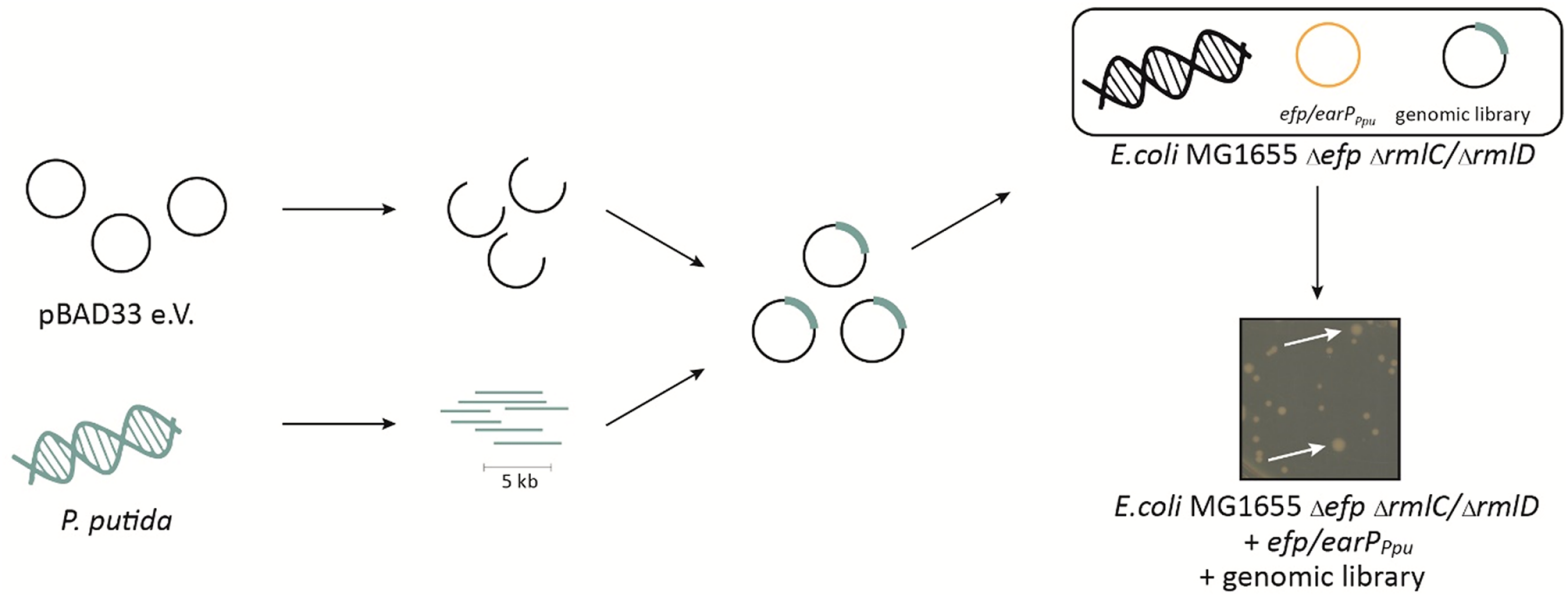
Screening strategy for the identification dTDP-l-Rha biosynthesis genes in *P. putida.* Chromosomal DNA of *P. putida* (green) was fragmented by restriction digestion. Fragments with an average size of 5 kb were then ligated into the arabinose inducible vector (pBAD33). The resulting library was transformed into an *E. coli* Δ*efp* P_cadBA_::*lacZ* reporter strain that concomitantly lacks either *rmlC* or *rmlD* (Δ*rmlC*/ Δ*rmlD*) and additionally encodes *earP*_*Ppu*_ and *efp*_*Ppu*_ (orange) of *P. putida* in trans. Genes cross-complementing Δ*rmlC* and Δ*rmlD* recover the impaired growth phenotype and can be selected by size (white arrows).

The library was constructed by partial restriction digestion of the *P. putida* genome with the *dam* and CpG methylation insensitive enzyme *Stu*I (NEB) (Fig. 3). The average fragment size was set to 5 kb to ensure that at least one gene was completely covered (average gene size: 1.132 kbp). These were cloned into *Sma*I linearized pBAD33, which allows for high-level expression by induction of the P_*BAD*_ promoter with l-arabinose^32^. Transformation of *E. coli* DH10B cells with the library revealed ~ 430,000 clones indicating an around 350-fold coverage of the *P. putida* KT2440 genome (total length 6.18187 Mbp).

**Figure 4:**
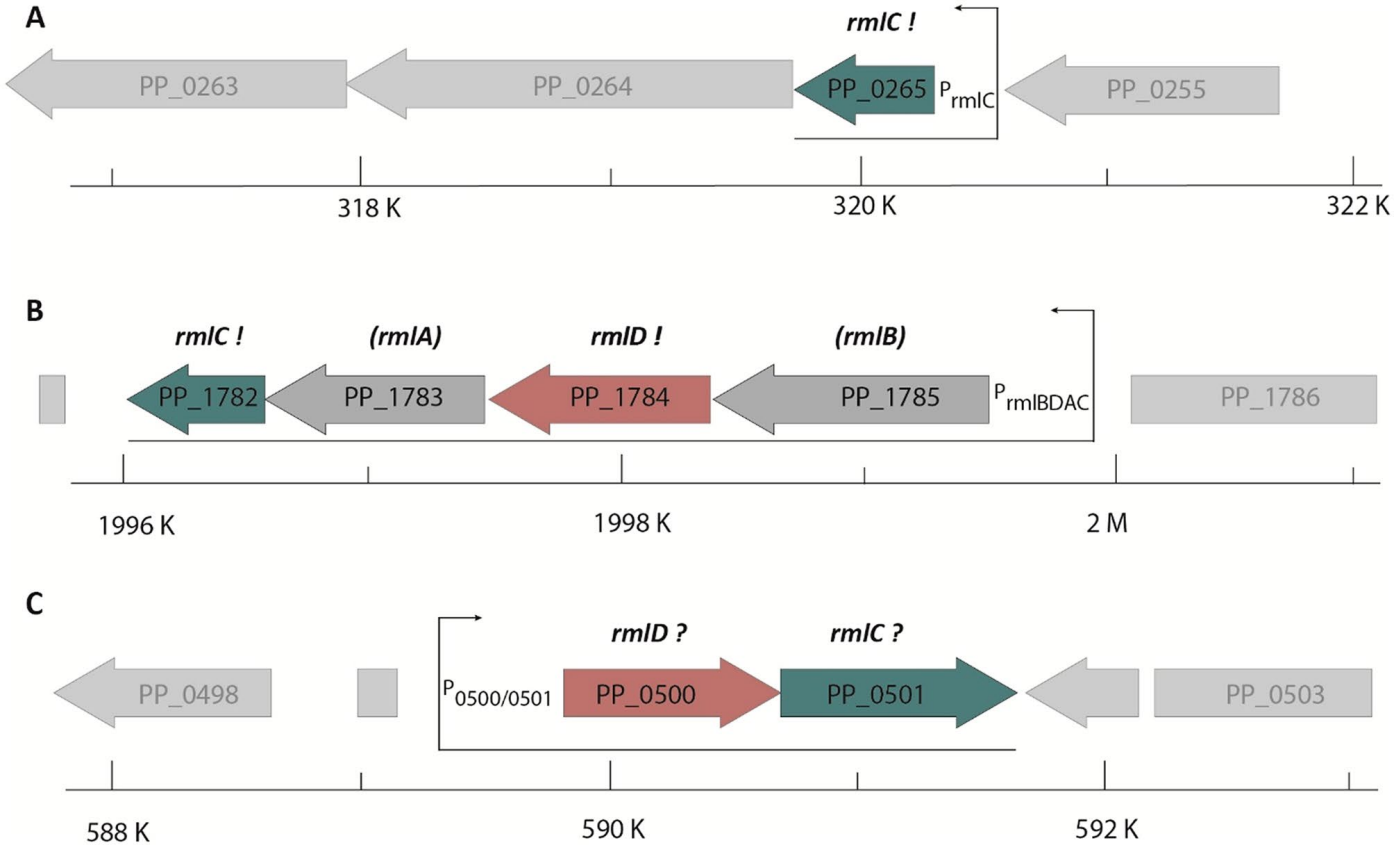
Genomic organization of *rmlC* and *rmlD* candidate genes in *P. putida*. (**A**) PP_0265 gene region, (**B**) PP_1782_PP_1784 gene region. (**C**) PP_0500 and PP_0501 gene region. Putative (?) or validated (!) homologs/analogs of *rmlC* and *rmlD* are shown in green and red respectively. Bottom: position within *P. putida* genome. Arrows indicate monocistrons. The scale indicates the position within the *P. putida* genome.

Next, we transformed *E. coli* Δ*efp* Δ*rmlC* + *efp*/*earP*_*Ppu*_ with the library and cultivated the cells in LB (lysogeny broth)^33,34^ containing 0.2% l-arabinose. Considering duplication times (Fig. 2B) and genome coverage, we expect *rmlC* copies to accumulate already to a single-digit percentage of the total population within latest two days (= ~ 16 generations with mutant growth phenotype and ~ 32 for wild-type phenotype), even under unfavorable circumstances. Indeed, when plating the second overnight culture on LB agar we obtained colonies of two different sizes. Consequently, we isolated plasmids from 16 large clones and sequencing identified 12 times PP_1782 and four times PP_0265 as the insert. PP_0265 (from now on *rmlC2*/RmlC2) resides next to genes encoding a putative two component signal-transduction system (Fig. 4A). PP_1782 (from now on *rmlC1*/RmlC1) on the other hand is the last of four genes in a putative dTDP-l-Rha biosynthesis operon PP_1785-PP_1782 (Fig. 4B). To substantiate our hypothesis on the dTDP-l-Rha biosynthetic operon, we conducted a second library screen with *E. coli* cells now lacking *rmlD* in addition to *efp* (Δ*efp* Δ*rmlD* + *efp*/*earP*_*Ppu*_) instead of *rmlC.* With this strain we exclusively enriched clones harboring a copy of PP_1784 (from now on *rmlD*/RmlD), a homolog of *E. coli* RmlD. Thus, we provide experimental evidence that PP_1785-PP_1782 form a *rmlBDAC1* operon in *P. putida* KT2440 and further identified a second gene encoding for an dTDP-4-dehydrorhamnose 3,5-epimerases—RmlC2.

### PP_0265/PP_1782 and PP_1784 are dTDP-4-dehydrorhamnose 3,5-epimerases and dTDP-4-dehydrorhamnose reductase, respectively

Our library screen was complemented by database mining and a homology search. In addition to *rmlC1* and *rmlC2,* we found PP_0501 being annotated as nucleoside diphosphate sugar epimerase of unknown specificity and as such might function as further dTDP-4-dehydrorhamnose 3,5-epimerase (String^35,36^, Pfam^37^, Uniprot^38^, Metacyc^39^ database). However, while RmlC1 & RmlC2 are highly homologous to each other (64% identity), PP_0501 shares no similarities at the sequence level. Nonetheless and in addition to its annotated function PP_0501 forms an operon with a putative dTDP-4-dehydrorhamnose reductase gene, PP_0500^35,36,40^ (Fig. 4C). This protein, on the contrary, shares similarities with RmlD both at the sequence level (29% identity) as well as structurally.

**Figure 5:**
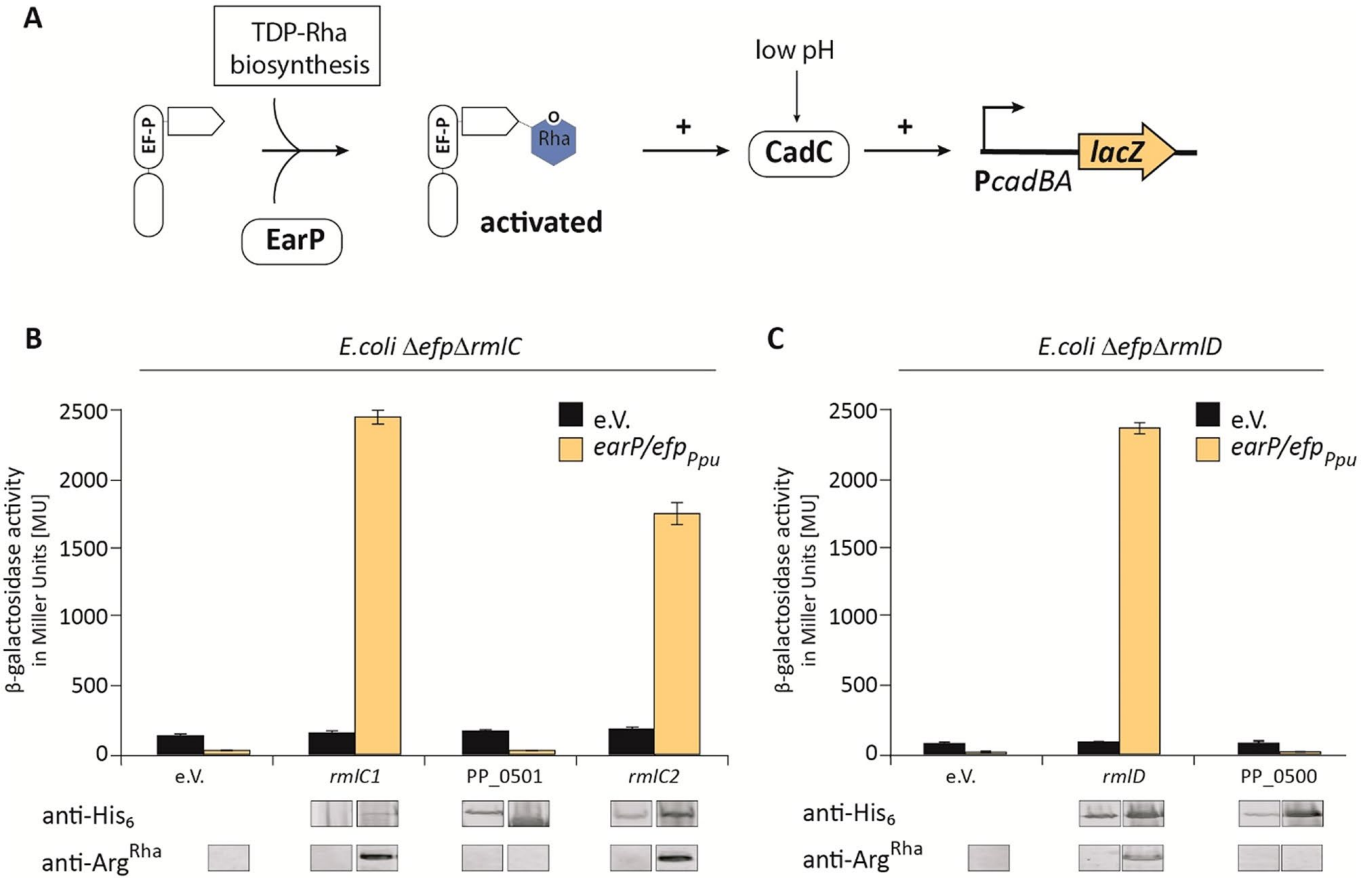
Analysis of in vivo activities of activated EF-P in dTDP-l-Rha biosynthesis deletion strains. (**A**) β-Galactosidase reporter assay. The assay is based on the lysine decarboxylase acid stress response of *E. coli*, the CadABC module^43^. At low pH, the transcriptional activator CadC activates the promoter of its two downstream genes (P_*cadBA*_) thereby inducing the expression of *lacZ* in an *E. coli* MG1655 P_*cadBA*_::*lacZ* strain. Proper translation of CadC is dependent on the presence of EF-P which is activated by mono-rhamnosylation, a reaction catalyzed by the glycosyltransferase EarP using dTDP-l-Rha (blue) as substrate. Thus β-galactosidase activity can be taken as an indirect readout for functional dTDP-l-Rha biosynthesis. (**B**,**C**) Functionalities of RmlC1, RmlC2, RmlD, PP_0500 and PP_0501 were determined by measuring the β-galactosidase activities of *E. coli* MG1655 P_*cadBA*_::*lacZ* Δ*efp* Δ*rmlC* (**B**)*/*Δ*rmlD* (**C**) with heterologous expression of a candidate gene from the pBAD33 vector. The empty vector (e.V.) was included as negative control. Additionally, all strains encoded the *earP/efp*_*Ppu*_ operon in trans, being encoded from pBBR MCS2 vectors (grey bars) expressed from the native promoter. Again, the corresponding empty vector served as control (black bars). All strains were grown o/n in LB pH 5.8 and activity is given in Miller Units (MU). Means of three independent measurements are shown. Standard deviations from three independent experiments were determined. Bottom: Western blot analysis of o/n cultures *E. coli* depicted in (**B**) and (**C**)*.* Rhamnosylated *EF-P*_*Ppu*_ (EF-P^Rha^) was detected using 0.25 µg/ml anti-Arg^Rha^. Expression of candidate genes was verified using 0.1 µg/ml anti-His_6_. Full-length Western Blots and corresponding SDS-gels are depicted in Fig. S3.

To test the putative role of PP_0500 and PP_0501 in dTDP-l-Rha biosynthesis we made again benefit of EarP mediated activation of *P. putida* EF-P and its functionality in *E. coli.* Hence, we cloned the two genes into pBAD33 simultaneously adding a His_6_-tag coding sequence for immunodetection in order to ensure proper protein production (Fig. 5). *rmlC1*, *rmlC2* and *rmlD* were also included in the study. The resulting plasmids pBAD33-*rmlC1*, pBAD33-*rmlC2*, pBAD33-PP_0501 as well as pBAD33-*rmlD* and pBAD33-PP_0500 were introduced into *E. coli* Δ*efp* Δ*rmlC* + *efp/earP*_*Ppu*_ and Δ*efp* Δ*rmlD* + *efp/earP*_*Ppu*_, respectively. Of note, these are reporter strains in which EF-P functionality is coupled to LacZ expression (Fig. 5A). Whereas β-galactosidase activity is low in cell with an incomplete dTDP-l-Rha biosynthesis pathway, introduction of either *rmlC1, rmlC2* (Fig. 5B) or *rmlD* (Fig. 5C) into the respective mutant strains led to a significant increase. By contrast, neither PP_0500 nor PP_0501 were able to rescue the Δ*efp*_*Eco*_ mutant phenotype.

In parallel we analyzed the rhamnosylation status of EF-P_*Ppu*_ utilizing anti-rhamnosylarginine specific antibodies (anti-Arg^Rha^)^21,41,42^. Immunodetection of EF-P_*Ppu*_ rhamnosylation matched with the reporter expression levels on the one hand confirming the enzymatic activities of RmlC1, RmlC2 and RmlD as dTDP-4-dehydrorhamnose 3,5-epimerase and dTDP-4-dehydrorhamnose reductase, respectively. On the other hand, they falsify speculation and database annotations that attribute PP_0500 and PP_0501 a function in dTDP-l-Rha biosynthesis (String^35,36^, Pfam^37^, Uniprot^38^, Metacyc^39^ database).

## Discussion

In the scope of this study, we have investigated the dTDP-l-Rha pathway of *P. putida* KT2440 with a focus on the epimerization of dTDP-4-keto-6-deoxy-d-glucose. Combining an unbiased approach and utilizing a genomic library, we identified two paralogous proteins RmlC1 and RmlC2. Duplication of *rmlC* is not restricted to *P. putida* KT2440 but certain other pseudomonads such as *P. monteilii*, *P. fulva*, *P. plecoglossicida* or *P. asiatica* harbor also two gene copies. In fact, functional redundancy in the dTDP-l-Rha biosynthesis pathway is nothing unusual. As an example, the two enzymes RffH and RffG of *E. coli* are paralogous to RmlA and RmlB, respectively^13^. Such duplications may be useful, e.g., to compensate for bottleneck reactions in the dTDP-l-Rha biosynthesis^44^. Such bottlenecks can occur at different stages as the pathway is not only utilized to ultimately generate dTDP-l-Rha. Specifically, dTDP-4-keto-6-deoxy-d-glucose is also a precursor of dTDP-3-acetamido-α-d-fucose^45^ and TDP-d-viosamine^46^ which are found as part of the glycan pattern in *P. syringae*^47^. Similarly, the two paralogs RmlC1 and RmlC2 in *P. putida* KT2440 might serve as starting point of similar but so far unknown reactions. Moreover, gene duplications open the gate for regulated expression in turn allowing the precise adjustment of the desired ratio of distinct NDP-sugars depending on parts of the dTDP-l-Rha biosynthesis pathway. It would also allow for the accumulation of educts or products of the preceding reactions such as dTDP-glucose and Glc-1P. Notably, whereas *rmlC1* is part of an operon in which presumably the full dTDP-l-Rha pathway is encoded, the *rmlC2* resides in the vicinity of two genes encoding a two-component system (TCS) of thus far unknown function. Based on the predicated domain composition, this specific TCS presumably transduces external signals into gene transcription. One might therefore speculate on regulated expression of *rmlC2* according to the environmental conditions.

While our genomic library revealed two RmlC paralogs in *P. putida* database mining indicated a further enzyme with similar activity PP_0501. However, our in vivo rhamnosylation assay disproved the initial hypothesis. Notably, the UDP-*N*-acetylglucosamine C4-epimerase PelX from *P. protegens* Pf-5 is structurally the closest homolog (identity 67%)^48^. PelX is involved in the biosynthesis of the GalNAc-rich bacterial polysaccharidepolysaccharide Pel, that is essential for pellicle biofilm formation^48,49^. One can hence hypothesize, that PP_0501 and the adjacent putative reductase PP_0500 might be involved in that pathway, instead.

## Material and methods

### Bacterial strains and growth condition

All strains and plasmids used in this study are listed and described in Table 1. *E. coli* cells were grown in Miller modified Lysogeny Broth (LB)^33,34^ at 37 °C aerobically under agitation, if not indicated otherwise. LB agar plates contained 1.5% agar. Mean diameters were measured from 20 colonies from 2 different LB agar plates from of the respective strain after incubation at 37 °C for 16 h. Growth measurements were conducted in 96 well plates. Therefore, 200 µl LB was inoculated with o/n cultures at an OD_600_ 0.001. OD_600_ was monitored in 10-min intervals for 12 h in a Tecan Spark with 240 rpm at 37 °C. The medium was supplemented with antibiotics at the following concentrations: 50 µg/ml kanamycin sulfate and 30 µg/ml chloramphenicol. Plasmids carrying the P_BAD_ promoter^32^ were induced with l-arabinose at a final concentration of 0.2% (w/v).

**Table 1:** Plasmids and strains used in this study.

	Feature/genotype	References
**Plasmid**		
pBAD33	CamR-cassette, p15A origin, araC coding sequence, ara operator	^32^
pBBR1MCS2	KanR-cassette, pBBR origin of replication, *oriT*	^50^
pBAD33_*rmlC1*	CamR-cassette, arabinose inducible expression of RmlC1	This study
pBAD33_*rmlD*	CamR-cassette, arabinose inducible expression of RmlD	This study
pBAD33_PP_0265	CamR-cassette, arabinose inducible expression of PP_0265	This study
pBAD33_PP_0500	CamR-cassette, arabinose inducible expression of PP_0500	This study
pBAD33_PP_0501	CamR-cassette, arabinose inducible expression of PP_0501	This study
pBBR1MCS2_*earP_efp*	KanR-cassette, *earP* and *efp* including the P_*earP*_ native operon promoter	^6,21^
**Strain**		
*E. coli* DH5αλpir	F-λ-endA1 glnV44 thi-1 recA1 relA1 gyrA96 deoR nupG Φ80dlacZΔM15 Δ(lacZYA-argF) U169, hsdR17(rK− mK+)	^51^
*E. coli* DH10B	F– *mcrA* Δ(*mrr-hsdRMS-mcrBC*) φ80*lacZ*ΔM15 Δ*lacX74 recA1 endA1 araD139* Δ (*ara-leu*)7697 *galU galK* λ– *rpsL*(Str^R^) *nupG*	^52^
*E. coli* MG1655	K-12 F− λ− ilvG− rfb-50 rph-1	^53^
*E. coli* PcadBA::*lacZ* Δ*efp*	MG1655 PcadBA::*lacZ* Δ(cadBA) Δ*efp*	^26^
*E. coli* PcadBA::*lacZ* Δ*efp* Δ*rmlC*	MG1655 PcadBA::*lacZ* Δ(cadBA) Δ*efp* Δ*rmlC*	^26^
*E.coli* PcadBA::*lacZ* Δ*efp* Δ*rmlD*	MG1655 PcadBA::*lacZ* Δ(cadBA) Δ*efp* Δ*rmlD*	^26^

### Molecular biology methods

Oligonucleotides used in this study are listed and described in the Supplementary Table S1. Plasmid DNA was isolated using the Hi Yield Plasmid Mini Kit from Süd Laborbedarf according to manufacturer’s instructions. DNA fragments were purified from agarose gels using the Hi Yield Gel/PCR DNA fragment extraction kit from Süd Laborbedarf. All restriction enzymes, DNA modifying enzymes and the Q5 high fidelity DNA polymerase for PCR amplification were purchased from New England BioLabs and used according to manufacturer’s instructions.

### Genomic library

The genomic DNA (gDNA) was isolated from 50 ml o/n culture of *P putida* KT2440 according to the protocol described in reference^54^. Further purification was achieved using Phase Lock Gel (QuantaBio) with Phenol–Chloroform. After the centrifugation, isopropanol precipitation was repeated. The pellet was resuspended in water, the final amount was 60 µg DNA.

Plasmid DNA was purified as described in “Molecular biology methods” from 12 ml *E. coli* DH5α cells. The plasmid DNA was diluted in water, the final amount was 10 µg DNA.

The library was constructed using *Sma*I (pBAD33 vector) and *Stu*I (gDNA) for digestion resulting in an average size of 5 kb per insert (Bionexus, Inc.). After ligation, the plasmids were transformed into *E. coli* DH10 B (Lucigen). Quality control was done by restriction digest of library clones with *BamH*I. All restriction enzymes were produced by New England Biolabs, Frankfurt. The library was reisolated from *E. coli* DH10B as described in “Molecular biology methods” and transferred into corresponding reporter strains.

### Bioinformatic tools

The multiple sequence alignment was generated using NCBI BLAST^55,56^ and Clustal Omega^57^. Candidate homologues were identified and analysed using String^35,36^, Pfam^37^, Uniprot^38^, Metacyc^39^ databases. Protein structures were predicted using Phyrre2^58^. Illustrations were generated with UCSF Chimera^59^.

### Β-Galactosidase assay

*E. coli* MG1655 P_cadBA_::*lacZ* Δ*efp* Δ*rmlC/*Δ*rmlD* expressing lacZ under the control of the cadBA promoter were grown in buffered LB (pH 5.8) overnight (o/n) and harvested by centrifugation. β-Galactosidase activities were determined as described in reference in biological triplicates and are given in Miller units (MU)^60^. Standard deviations from three independent experiments were determined.

### SDS-PAGE and western blotting

Electrophoretic separation of proteins was carried out using 12.5% SDS-PAGE as described by Laemmli^61^. Separated proteins were visualized in gel using 0.5% (vol/vol) 2-2-2-trichloroethanol^62^ and detected within a Gel Doc EZ gel documentation system (Bio-Rad). The proteins were transferred onto a nitrocellulose membrane by vertical Western blotting (4 °C). Antigens were detected using 0.1 g/ml anti-His_6_ tag (Abcam, Inc.) or 0.25 g/ml of anti-Arg^Rha^^41^. Primary antibodies (rabbit) were the targeted using 0.1 µg/ml anti-rabbit IgG (IRDye 680RD) (donkey) antibodies (Abcam). Target proteins were visualized via Odyssey CLx Imaging System (LI-COR, Inc).

## Supplementary Information

Supplementary Information.
