# Supplementary material for: Two RmlC homologs catalyze dTDP-4-keto-6-deoxy-d-glucose epimerization in Pseudomonas putida KT2440
Source: Sci Rep. 2021 Jun 7;11:11991. doi: 10.1038/s41598-021-91421-x (PMC8184846; doi:10.1038/s41598-021-91421-x)
Supplement: Supplementary file 1 — Supplementary Information. [file 41598_2021_91421_MOESM1_ESM.docx]

Supplement Figures


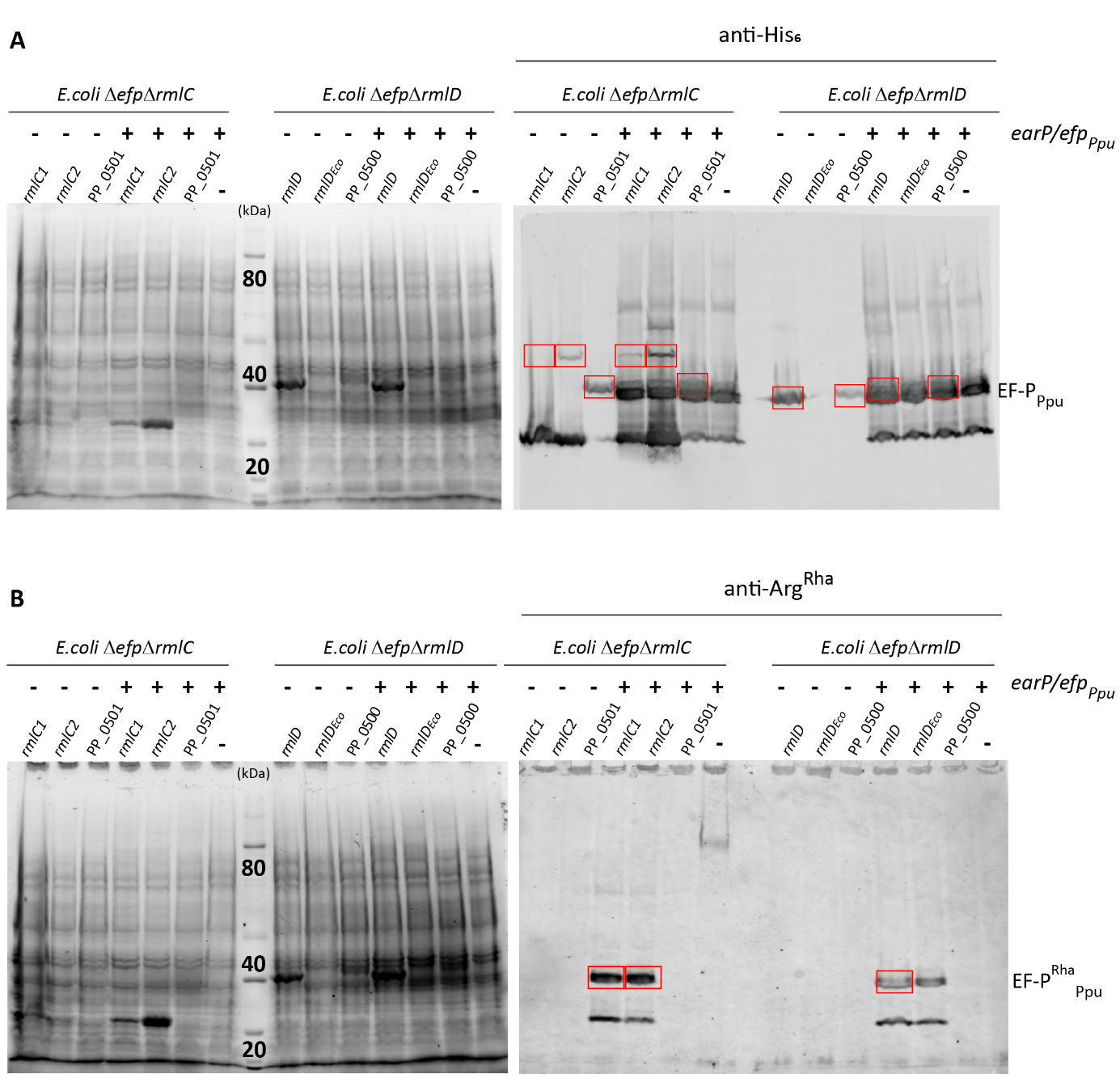


Fig. S1. **Analysis of activated EF-P in TDP-Rha biosynthesis deletion strains *via* western blot.** Functionalities of RmlC1, RmlC2, RmlD, PP_0500 and PP_0501 were determined by western blot in *E. coli* MG1655 P*_cadBA_*::*lacZ* Δ*efp* Δ*rmlC/*Δ*rmlD* with heterologous expression of a candidate gene from the pBAD33 vector. The empty vector (**-**) was included as negative control. Additionally, all strains encoded the *earP/efp_Ppu_* operon in trans, being encoded from pBBR MCS2 vectors expressed from the native promoter (**+**). Again, the corresponding empty vector served as control (**-**). All strains were grown o/n in LB pH 5.8. **A**) Expression of candidate genes was verified using 0.1 µg/ml anti-His_6_. **B**) Rhamnosylated *EF-P_Ppu_* (EF-P^Rha^) was detected using 0.25 µg/ml anti-Arg^Rha^.

Table S1: Oligonucleotides used in this study.

| Identifier | Oligonucleotide | Sequence (5'-3') | Restriction site |
| --- | --- | --- | --- |
| P1 | Seq33_fw | GGCGTCACACTTTGCTATGC |  |
| P2 | pBAD-HisA_rev | CAGTTCCCTACTCTCGCATG |  |
| P3 | SacI_rmlC1_fw | TTCGAGCTCGGAATTGTGAGCGGATAACAATTCCCCTCTAGAAATAATTTTGTTTAACTTTAAGAAGGAGATATACATATGCAAGCCATTCCGCTGGATATCC | SacI |
| P4 | SalI_rmlC1_rev | AGGTCGACTCTAGATTAGTGATGGTGATGGTGATGGCTGCCGTCGAACAGTTCGGCGTC | SalI |
| P5 | SacI_PP_rmlD_fw | GCCGAGCTCTGGCGACATATAAGACACTAAGGAGGTATTTTATGGTGAAAATCCTGCTGTTGGGGAAAA | SacI |
| P6 | SalI_PP_rmlD_rev | GGTCGACTCTAGATTAGTGATGGTGATGGTGATGGCTGCCCTGGATTTCGTCCAGCATACG | SalI |
| P7 | Sacl_PP_0265_fw | GCCGAGCTCGCGGCGAAAGCCGCCCCAGCAATCACCCTAAGGAGGTATTTTATGAACATCATCCCTACAGCAATCC | SacI |
| P8 | SalI_PP_0265_rev | GGTCGACTCTAGATTAGTGATGGTGATGGTGATGGCTGCCAGGCAGCACTTCCGCCTC | SalI |
| P9 | SacI_sRBS_PP_0500-fw | TTCGAGCTCGAGTACTCGGATTTCGATTGGTTTAAGGAGGAAAAATATGCGTATGCGCCTAATGCTG | SacI |
| P10 | XbaI_PP_500_GS-His6-rev | CTCTAGATTAGTGATGGTGATGGTGATGGCTGCCGCCATGGCGGTAGAACCGG | XbaI |
| P11 | SacI_sRBS_PP_0501-fw | TTCGAGCTCATTCTTCTACGGATTACATAAGGAGGTTTTTTATGGCTGACGCCCCCATCC | SacI |
| P12 | XbaI_PP_501_GS-His6-rev | CTCTAGATTAGTGATGGTGATGGTGATGGCTGCCTTTACCGAGAAGCTTTGTC | XbaI |
